# Hazan J; Liu KY; Howard R. Clarity AD open-label extension data do not robustly confirm disease course modification by lecanemab in ApoE4 heterozygotes and non-carriers. # TJPAD-D-26-00165

**DOI:** 10.1016/j.tjpad.2026.100588

**Published:** 2026-05-06

**Authors:** Lutz Froelich

**Affiliations:** Medical Faculty Mannheim, University of Heidelberg, Central Institute of Mental Health, Mannheim, Germany

We thank Dr. Hazan and colleagues for their interest in our article [1] and for providing their feedback. In our recent manuscript, we presented efficacy and safety results in this subpopulation of Clarity AD, which was expected to yield similar efficacy as the overall population, but less ARIA, since ApoE ε4 homozygotes are more likely to experience ARIA than ApoE ε4 heterozygotes or non-carriers. We included data from the randomized, double blind phase of Clarity AD as well as open-label extension (OLE) data. As noted in our paper, this subgroup analysis of the original Clarity AD data was not pre-specified, but was requested by the European authorities (MHRA and EMA) as part of the submission for market authorization approval. The results in our paper show that lecanemab significantly reduced clinical decline on CDR-SB at 18 months compared to placebo in the ApoEε4 heterozygotes or non-carriers subgroup. Secondary endpoint results were consistent with the primary endpoint, including amyloid PET, ADAS-Cog14, ADCS-MCI-ADL, and HRQoL assessments. In the analysis subgroup, the most common adverse reactions for lecanemab were infusion-related reaction (26%), ARIA-H (13%), fall (11%), headache (11%), and ARIA-E (9%). We also present health-related quality of life data as well as results from the Clarity AD open-label extension (up to 36 months), which suggest an expanding benefit over time for lecanemab long-term treatment.

The criticism on our paper from Dr. Hazan and colleagues focus mainly on the ‘claims’ made from the OLE results. However, we clearly lay out the limitations on these data and do not present statistical analyses on these data. They argue that rigorous and well-designed randomized, controlled trials are the preferred approach to answer key questions on topics such as disease modification and importance of starting treatment early. On this topic we completely agree with Dr. Hazan and colleagues and do not believe there is a dispute in the broader scientific community. However, there is value in publishing data from OLE studies as we have done in our paper. OLE trials have clinical and scientific value in providing additional data on long-term safety and effectiveness data beyond the time for the primary efficacy outcome evaluation. Systematic and rigorous long-term clinical and safety data from OLE studies extend and supplement the results of the randomized controlled trials. Regulatory health authorities generally require these studies as part of the evaluation process of potential new treatments, consistent with International Council for Harmonization (ICH) of Technical Requirements for Pharmaceuticals for Human Use guidelines [2]. Publication of these results is intended to inform clinicians, AD patients, and care partners.

In our paper, we very clearly lay out the study design and associated methods, we state that all the analyses were descriptive statistics, we provide a transparent accounting of the results, and we extensively review the limitations of the study. When analyses are not pre-specified, they are noted in the manuscript. We included a prospectively defined historical control to enhance the analysis and increase the hypothesis-generating potential of our results. We appreciate the suggestions for additional analyses from Dr. Hazan and colleagues. However, given the limitations of the data, we do not think that any further post-hoc analyses, e.g. between group differences or 95% confidence intervals for the continuous and delayed lecanemab groups would add to the value of our current analyses. As noted in the paper, the data is available upon reasonable request.

In conclusion, there is no argument that trials with rigorous designs should be conducted to definitively answer specific questions of importance, as suggested by Hazan et al. However, we reiterate that the results from OLE studies are important and can add to our overall understanding in the field. With the methods and associated limitations clearly defined, as we have done, our intention is for well-informed readers to objectively interpret the meaning and impact of the results. We are happy to have published our results, which taken together with all available Clarity AD publications help to better define and improve our understanding of the overall safety/efficacy profile for lecanemab.

## Declaration of the use of generative AI and AI-assisted technologies in scientific writing and in figures, images and artwork

AI was not used to generate any facet of this manuscript.

## CRediT authorship contribution statement

**Lutz Froelich:** Conceptualization, Formal analysis, Investigation, Supervision, Writing – original draft, Writing – review & editing.

## Declaration of competing interest

Lutz Froelich reports a relationship with Anavex, Avanir/Otsuka, Biogen, BioVie, Bristol-Myers-Squibb, DerCampus, Eisai, Eli Lilly, FOMF, Araclon/Grifols, Janssen Cilag, Johnson&Johnson, Medical Tribune, Medfora, Medscape, Neurimmune, Neuroscios, Noselab, NovoNordisk, PharmatrophiX, Hoffmann-LaRoche, TauRX, Schwabe, StreamUp, Vivoryon that includes: personal consulting fees.
